# Proteasome Inhibitors Induce Apoptosis in Ex Vivo Cells of T-Cell Prolymphocytic Leukemia

**DOI:** 10.3390/ijms252413573

**Published:** 2024-12-18

**Authors:** Vanessa Rebecca Gasparini, Elisa Rampazzo, Gregorio Barilà, Alessia Buratin, Elena Buson, Giulia Calabretto, Cristina Vicenzetto, Silvia Orsi, Alessia Tonini, Antonella Teramo, Livio Trentin, Monica Facco, Gianpietro Semenzato, Stefania Bortoluzzi, Renato Zambello

**Affiliations:** 1 Hematology Section, Department of Medicine, Hematology and Clinical Immunology Branch, University of Padova, 35122 Padova, Italy; vanessarebecca.gasparini@gmail.com (V.R.G.); elisa.rampazzo.3@phd.unipd.it (E.R.); elena.buson.1@studenti.unipd.it (E.B.); giulia.calabretto@gmail.com (G.C.); alessia.tonini@unipd.it (A.T.); antonella.teramo@unipd.it (A.T.); livio.trentin@unipd.it (L.T.); monica.facco@unipd.it (M.F.); r.zambello@unipd.it (R.Z.); 2 Veneto Institute of Molecular Medicine (VIMM), 35129 Padova, Italy; 3 Hematology Unit, San Bortolo Hospital, 36100 Vicenza, Italy; gregorio.barila@aulss8.veneto.it; 4 Department of Molecular Medicine, University of Padova, 35122 Padova, Italy; aburatin@carrerasresearch.org (A.B.); silvia.orsi@phd.unipd.it (S.O.); stefania.bortoluzzi@unipd.it (S.B.); 5 Cardiology, Department of Cardiac Thoracic Vascular Sciences and Public Health, University of Padova, 35122 Padova, Italy; cristina.vicenzetto@aopd.veneto.it; 6 Department of Biology, University of Padova, 35131 Padova, Italy

**Keywords:** leukemia, proteasome inhibitors, apoptosis

## Abstract

Finding an effective treatment for T-PLL patients remains a significant challenge. Alemtuzumab, currently the gold standard, is insufficient in managing the aggressiveness of the disease in the long term. Consequently, numerous efforts are underway to address this unmet clinical need. The rarity of the disease limits the ability to conduct robust clinical trials, making in silico, ex vivo, and in vivo drug screenings essential for designing new therapeutic strategies for T-PLL. We conducted a drug repurposing analysis based on T-PLL gene expression data and identified proteasome inhibitors (PIs) as a promising new class of compounds capable of reversing the T-PLL phenotype. Treatment of ex vivo T-PLL cells with Bortezomib and Carfilzomib, two PI compounds, supported this hypothesis by demonstrating increased apoptosis in leukemic cells. The current lack of a suitable in vitro model for the study of T-PLL prompted us to perform similar experiments in the SUP-T11 cell line, validating its potential by showing an increased apoptotic rate. Taken together, these findings open new avenues for investigating the molecular mechanisms underlying the efficacy of PI in T-PLL and expand the spectrum of potential therapeutic strategies for this highly aggressive disease.

## 1. Introduction

T-cell prolymphocytic leukemia (T-PLL) is a rare and extremely aggressive disease characterized by the clonal expansion of mature CD4+ T-cells, leaving patients with limited therapeutic options and poor clinical outcomes [1]. The leukemic clone usually displays a post-thymic phenotype (CD2+/CD3+/CD5+/CD7+), with the majority of cases being CD4+CD8±, although CD4-−CD8+ expansions have been rarely reported (representing <15% of total cases) [2,3,4]. The median age at disease onset is 65 years, with a median overall survival (OS) from diagnosis of <3 years [1,2,5]. The disease may present as indolent in 20% of patients, but it usually progresses into the aggressive form within two years [6]. T-PLL patients typically exhibit marked lymphocytosis (>100 × 10^9^/L), hepatosplenomegaly, and generalized lymphadenopathy [5,7]. The major criteria required for the diagnosis of T-PLL are the identification of a T-cell clone of at least 5 × 10^9^/L cells in the peripheral blood or bone marrow, along with the presence of abnormalities of 14q32 or Xq28 or expression of *MTCP1* or *TCL1A* or *B* [1,2].

The molecular landscape of most T-PLL patients displays recurrence of a complex karyotype, with the overexpression of the proto-oncogene *TCL1A/B* [1,8], and several activating mutations identified in numerous genes, including those within the *IL2RG-JAK1-JAK3-STAT5B* pathway [4,9,10,11,12,13]. Other key mutations have been discovered in driver genes involved in cell cycle regulation and apoptosis (*ATM* and *MYC*), as well as those implicated in epigenetic regulation, like *EZH2*, contributing to the dysregulated cell proliferation and survival of leukemic cells [1,14].

Given that CD52 expression is typically high at disease onset, the current gold standard for the treatment of T-PLL relies on the anti-CD52 monoclonal antibody, Alemtuzumab [5,10,15,16]. The introduction of this immunotherapeutic approach has proven efficacy in the clinical management of 80% of patients. However, it fails to induce long-term remission unless consolidated with hematopoietic stem cell transplantation. Without this consolidation, the median OS remains unacceptably short [1,5]. Furthermore, the feasibility of consolidation treatment with allogeneic stem cell transplantation is limited by the advanced age of the patients at diagnosis and by the presence of comorbidities [17,18]. Therefore, there is an urgent need for new treatment options, and both research studies and clinical trials are actively underway in the effort to treat this incurable disease [19,20,21,22].

Drug sensitivity and resistance tests performed on T-PLL samples [4] indicated PI3K/AKT/mTOR inhibitors [4], histone deacetylase inhibitors [4,23], inhibitors of Cyclin-dependent Kinases (CDKs), and the BCL-2 inhibitor Venetoclax [24] as the most promising compounds [1,5]. Other studies focused on reconstituting p53 [25], which is often inactivated in T-PLL due to mutations in *ATM*, an upstream gene in the signaling pathway responsible for p53 activation. Reactivation of p53 has been attempted through the use of MDM2 antagonists, which in combination with HDAC inhibitors were able to induce apoptosis in T-PLL cells [5,10]. These compounds have been tested as single agents and in combination with other FDA-approved [24,26,27,28,29,30,31] drugs in the clinical management of T-PLL patients. More recently, a new study confirmed the efficacy of Cladribine, Romidepsin, Venetoclax, and Idasanutlin in inducing apoptosis of T-PLL cells both in vitro and in vivo [32]. The lack of personalized treatment for T-PLL patients highlights the need for a further understanding of the altered pathways in this disorder, which is essential for identifying innovative treatments that might ultimately improve patients’ outcomes.

In this study, we conducted a drug repurposing screening based on gene expression profiles of T-PLL patients, which identified proteasome inhibitors (PIs) as some of the most effective drugs. We also demonstrated the ability of these compounds to induce apoptosis in ex vivo T-PLL patients’ cells, suggesting that this class of inhibitors could represent a promising new strategy for the treatment of this disease. PIs could potentially be used in combination with other drugs already employed in clinical practice.

## 2. Results

### 2.1. Clinical and Molecular Features of the T-PLL Cohort

Our study included 17 cases diagnosed with T-PLL. The median age at diagnosis was 72 years without differences in gender. Other clinical parameters evaluated confirmed the presence of high white blood cell count (WBCs, 133 × 10^9^/L on average) associated with a striking lymphocytosis in almost all the cases. The diagnostic workflow included also cytofluorimetric analysis for the evaluation of the immunophenotype of the leukemic clone and the clonality assessment (Table 1). All patients recruited presented a unique clone comprising positivity for CD7 and CD4 surface markers, whereas the expression of other markers (i.e., CD25 and CD26) was variable within the cohort. In all the cases, clonality was established through TCR rearrangement analysis. Furthermore, the analysis of the TCR-Vbeta repertoire confirmed clonal expansion in half of the cases, defined by the presence of a discrete Vbeta expansion. In the other cases, the results were inconclusive because the kit used, although widely employed in diagnostic laboratories, covers only about 70% of the TCR-Vbeta repertoire, thus making it impossible to determine a definitive clone. Sanger sequencing performed on the hotspot regions of the two most recurrently mutated genes in T-PLL identified 35% of patients harboring mutations in *STAT5B* gene (with a prevalence of N642H variant) and 12% of patients with mutations in *JAK3* gene, with both patient groups harboring the M511I variant (Table 1).

### 2.2. Proteasome Inhibitors Are the Major Class of Compounds Potentially Capable of Reversing the T-PLL Phenotype

The main focus of this study was the identification of drugs that are already in use in the clinical management of other diseases and that might be repositioned for T-PLL.

From the profiling of 10 T-PLL patient samples and CD4+ T-cells sorted from 5 healthy donor samples, used as normal counterparts, a T-PLL signature consisting of the most dysregulated 300 genes (larger expression log fold change in the comparison) among those significantly differentially expressed (*q* < 0.05) in T-PLL was established.

Next, bioinformatic analyses were conducted to identify FDA-approved small-molecule compounds whose experimentally determined effects on gene expression in vitro are significantly correlated, either positively or negatively, with the T-PLL gene expression signature. Particularly, those found in the LINCS that are significantly negatively connected with an observed gene expression signature are interesting candidates for further experiments, since they have the potential to “revert the phenotype”, thus meaning that they could induce expression changes opposite to those observed in the disease.

**Figure 1:**
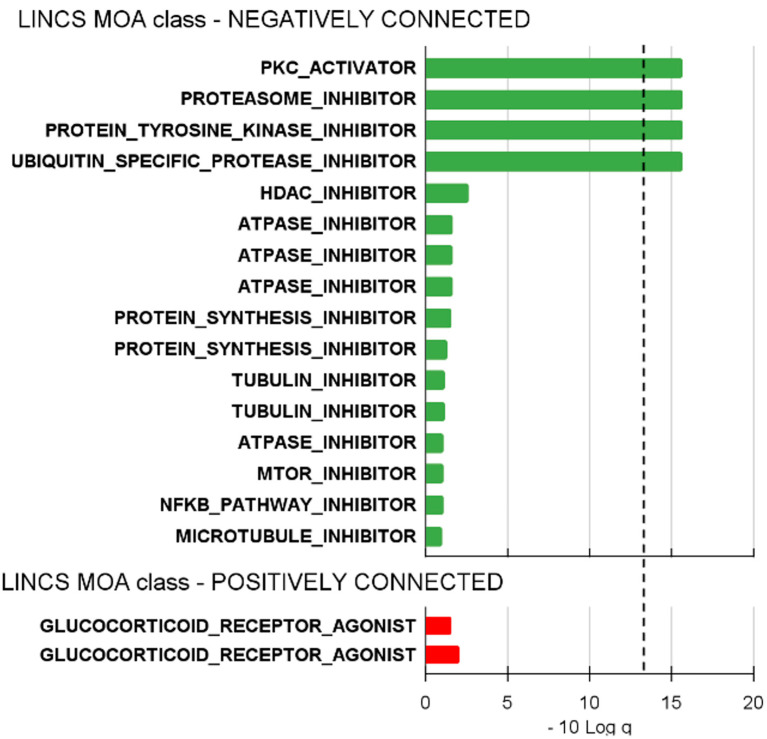
Proteasome inhibitors (PIs) as additional therapy for T-PLL. Barplot representing the significant level of association between drug LINCS (Library of Integrated Cellular Signatures, category Mechanism of Action) and T-PLL expression profile obtained by drug repurposing analysis to predict the compounds putatively most effective in T-PLL. Negatively connected signatures indicate compounds potentially able to revert the phenotype; association values over 1 are shown; values over the black line are statistically significant (*q*-value < 0.5). MOA: mechanism of action.

From our analysis, 18 MoA LINCS members were connected with the T-PLL signature. Four LINCS members significantly and negatively connected with the T-PLL signature were of particular interest for their potential to inhibit the leukemic progression and can be considered for future clinical use (Figure 1). Protein kinase C activators, proteasome inhibitors, protein tyrosine kinase inhibitors, and ubiquitin-specific protease inhibitors were detected as the most promising drugs with signatures significantly negatively connected with the T-PLL gene expression profile (Figure 1). Among these promising candidates, we selected the class of proteasome inhibitors for the in vitro and ex vivo experiments in the context of T-PLL. Thus, Bortezomib (PS-341), a reversible inhibitor of the 26S proteasome, and Carfilzomib (PX-171-007), an irreversible PI, were selected for experimental tests.

### 2.3. Evaluation of Apoptosis After Treatment with Bortezomib

**Figure 2:**
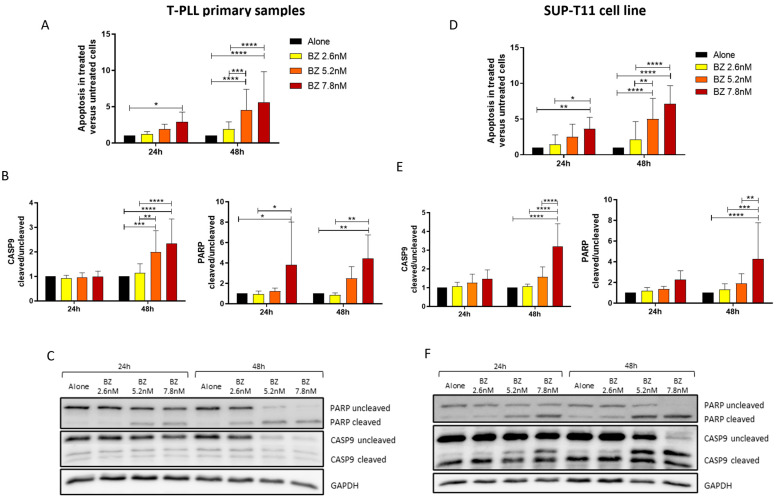
Evaluation of apoptosis after treatment with Bortezomib. (**A**,**D**) Ratio of apoptosis in treated over untreated (alone) condition in ex vivo T-PLL cells (**A**; *n* = 16) and SUP-T11 cell line (**D**; *n* = 11) after treatment with BZ for 24 and 48 h. The ratio of apoptosis was calculated as follows: Ratio of Apoptosis = Number of apoptotic cells (Annexin V+)/Total number of cells analyzed (live, apoptotic, and dead). This quantification was based on flow cytometry data, where apoptotic cells were identified using Annexin V/PI staining. The ratio reflects the proportion of apoptotic cells relative to the total cell population. (**B**,**E**) Densitometric analysis of PARP and CASP9 proteins in ex vivo T-PLL cells (**B**; *n* = 12) and SUP-T11 cell line (**E**; *n* = 11) after treatment with BZ up to 48 h. Protein cleavages were analyzed calculating the ratio between the cleaved and uncleaved fraction for both proteins; GAPDH was used as internal loading control. All analyses were normalized against the untreated (alone) condition. (**C**,**F**) Representative Western blotting images. BZ: Bortezomib; h: hours; PARP: Poly (ADP-ribose) polymerase; CASP9: Caspase 9; * *p* < 0.05; ** *p* < 0.01; *** *p* < 0.001; **** *p* < 0.0001 (data were analyzed by two-way ANOVA test).

Bortezomib was first tested in primary T-PLL samples (Table 1) with scalar concentration of the drug, and apoptosis was evaluated at 24 h and 48 h (Figure 2A). The cytofluorimetric analysis confirmed the prediction from the bioinformatic analysis since a significant increase in apoptosis, considered as the ratio between treated versus untreated condition, was observed in a dose- and time-dependent manner. In particular, the differential proportion of cell death was significant at 24 h for the concentration of 7.8 nM (*p* < 0.05) and at 48 h for 5.2 nM (*p* < 0.0001). Western blotting analysis that focused on the quantification of the CASP9 and PARP protein cleavages (Figure 2B) further supported the efficacy of the treatment with BZ in the primary samples. Accordingly, the upregulation of both protein cleavages was observed, thus confirming the commitment of the cell towards apoptosis. In detail, the concentration of 7.8 nM induced a significant cleavage of both CASP9 and PARP proteins at 48 h (*p* < 0.01). CASP9 cleavage was also increased for BZ 5.2 nM at 48 h (*p* < 0.001), while PARP cleavage was also significant at 24 h after BZ 5.2 nM treatment (*p* < 0.05). A representative picture of the Western blotting is presented in Figure 2C, where the reduction in the uncleaved protein can be appreciated in favor of the cleaved one.

Considering the promising results obtained in the primary cells, the same experiments were reproduced in the SUP-T11 cell line with the final aim of confirming PI efficacy and to investigate the potential use of this cell line as a putative model for the study of this rare disease whose samples can be difficult to obtain. As reported in Figure 2D–F, the results are in line with those previously obtained in the primary samples. A significant increase in apoptosis was observed at both 24 h and 48 h for the concentration of 7.8 nM (*p* < 0.01 and *p* < 0.0001, respectively) and at 48 h for the 5.2 nM concentration (*p* < 0.001; Figure 2D). Also, at the protein level, a significant increase in CASP9 and PARP cleavage was observed for the highest concentration of BZ at 48 h (*p* < 0.0001; Figure 2E). In contrast, in the SUP-T11 context, no significant CASP9 cleavage was observed for the 5.2 nM concentration at 24 h, nor was PARP cleavage at 24 h for the 7.8 nM concentration. Figure 2F reports the representative Western blotting picture of the protein cleavages in SUP-T11, where the increase in the uncleaved fraction is even more appreciable compared to the primary samples.

### 2.4. Evaluation of Apoptosis After Treatment with Carfilzomib

**Figure 3:**
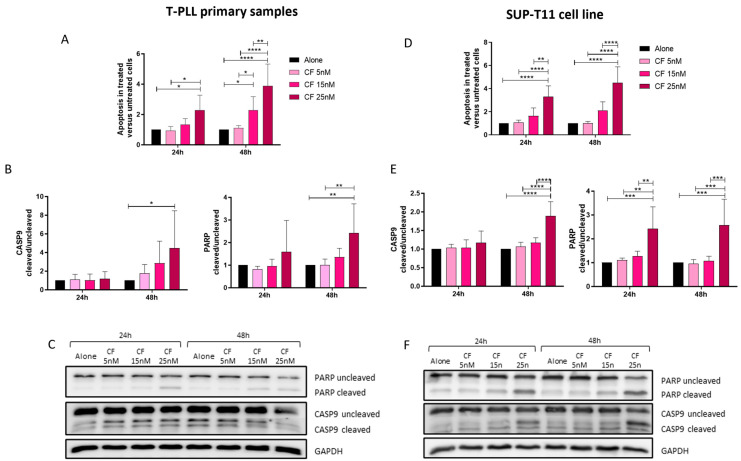
Evaluation of apoptosis after treatment with Carfilzomib. (**A**,**D**) Ratio of apoptosis in treated over untreated (alone) condition in ex vivo T-PLL cells (**A**; *n* = 6) and SUP-T11 cell line (**D**; *n* = 5) after treatment with CF for 24 and 48 h. The ratio of apoptosis was calculated as follows: Ratio of Apoptosis = Number of apoptotic cells (Annexin V+)/Total number of cells analyzed (live, apoptotic, and dead). This quantification was based on flow cytometry data, where apoptotic cells were identified using Annexin V/PI staining. The ratio reflects the proportion of apoptotic cells relative to the total cell population. (**B**,**E**) Densitometric analysis of PARP and CASP9 proteins in ex vivo T-PLL cells (**B**; *n* = 6) and SUP-T11 cell line (**E**; *n* = 5) after treatment with CF up to 48 h. Protein cleavages were analyzed calculating the ratio between the cleaved and uncleaved fraction for both proteins; GAPDH was used as internal loading control. All analyses were normalized against the untreated (alone) condition. (**C**,**F**) Representative Western blotting images. CF: Carfilzomib; h: hours; PARP: Poly (ADP-ribose) polymerase; CASP9: Caspase 9; * *p* < 0.05; ** *p* < 0.01; *** *p* < 0.001; **** *p* < 0.0001 (data were analyzed by two-way ANOVA test).

Then, T-PLL primary samples (Table 1) were treated with Carfilzomib, the irreversible PI, in order to analyze the effect of this different type of compound. An increase in the apoptotic ratio between treated and untreated condition was observed at 24 h (*p* < 0.05) and 48 h (*p* < 0.001) for the concentration of 25 nM and at 48 h (*p* < 0.05) for the lower concentration of 15 nM (Figure 3A), thus confirming that Carfilzomib also has the potential to induce leukemic cells towards cell death. Regarding the Western blotting analysis (Figure 3B,C), only the highest concentration of CF was able to increase the cleavage of CASP9 and PARP protein (*p* < 0.05 and *p* < 0.01, respectively), differently from BZ, where PARP cleavage was also observed at 24 h. A representative picture of the Western blotting analysis is shown in Figure 3C, where the PARP cleavage is more appreciable compared to the CASP9 one. Similar results were subsequently obtained in the SUP-T11 cell line (Figure 3D–F), investigated once more to observe the effect of this drug and to evaluate the use of the cell line as a model for further investigations. Moreover, a significant effect of CF at 25 nM was observed at 24 h and 48 h in respect to the control condition (both *p* < 0.0001), but also in respect to the other concentrations tested (*p*-value varying from <0.01 to <0.0001; Figure 3D), supporting the results observed in the primary cells. In contrast, in T-PLL cells, significant apoptosis was observed for 15 nM but not confirmed at the protein level. More pronounced protein dysregulation was observed with Western blotting analysis on SUP-T11 cells (Figure 3E) since CASP9 cleavage was significant at 48 h for CF at 25 nM compared to all the conditions (at least *p* < 0.001), and PARP cleavage with the same CF concentration was increased at both 24 h and 48 h (at least *p* < 0.01 in both cases). Accordingly, the representative Western blotting picture presented in Figure 3F clearly highlights the protein cleavages. Taking all the results together, it was possible to observe the efficacy of the treatment with the irreversible PI in the context of T-PLL and to confirm the reliability of the data in the in vitro model under investigation.

## 3. Discussion

Our research focused on the re-analysis of transcriptomic data available in the laboratory for a drug repurposing study aimed at identifying alternative therapeutic strategies for T-PLL. This screening, based on T-PLL patients’ gene expression profiles, identified proteasome inhibitors among the most effective drug candidates. Consequently, Bortezomib and Carfilzomib were selected as representative compounds of this class of drugs and were used to treat ex vivo T-PLL cells at varying dosages. The treatment of ex vivo T-PLL cells with Bortezomib and Carfilzomib demonstrated increased apoptosis of leukemic cells. Similar experiments were also conducted using the SUP-T11 cell line, further validating its potential as an in vitro model for the study of T-PLL, given the current lack of a suitable one.

Drug repurposing is particularly powerful in case of diseases such as T-PLL, where recruiting an adequate number of patients for a proper drug development trial is challenging due to the rarity of the disease. Moreover, the use of marketed approved drugs is both time- and cost-effective as their pharmacology, formulation, safety, and toxicity profiles are already established [33]. Bortezomib is currently used in the clinical management of plasma cell myeloma, relapsed/refractory mantle cell lymphoma, and acute lymphocytic leukemia [34,35,36], whereas Carfilzomib is effective in primary plasma cell leukemia, relapsed multiple myeloma, and relapsed/refractory acute lymphoblastic leukemia [37,38,39]. To date, only one study has tested BZ on ex vivo cells, which was performed several years ago on three T-PLL patients [40], while CF has yet to be investigated. Our culture experiments with PI confirmed the prediction of bioinformatic analysis, demonstrating a dose- and time-dependent increase in apoptosis following treatment with each compound. Cell death was observed at both cellular and protein levels, providing a starting point for further investigations into the efficacy of these drugs in T-PLL. These drugs could be used either alone or in association with recently proposed compounds such as HDAC, BCL2, and MDM2 inhibitors [5,10,23,24] to enhance their cytotoxic effect on T-PLL cells. To identify appropriate therapeutic options, the evaluation of specific markers (e.g., mutational status in hotspot genes) should be considered. Accordingly, T-PLL patients are characterized by the overexpression of *TCL1A/B* genes and other recurrent lesions in MTCP1, ATM, MYC, and STAT pathways, all of which play a crucial role in cell survival, proliferation, and oncogenic processes [41]. The limited number of T-PLL patients enrolled in this study (which took almost 10 years) underscores the need for a suitable in vitro model, which is still lacking. Previous studies have shown that the SUP-T11 cell line, derived from adult T-cell acute lymphoblastic leukemia and characterized by the overexpression of *TCL1A* gene, shares similar features with T-PLL cells [42,43]. Specifically, Cuesta-Mateos et al. [42] confirmed TCL1 protein overexpression and positivity for certain surface markers (i.e., CCR7, CD7, CD26), while Patil et al. [43] found a close molecular resemblance. For these reasons, the SUP-T11 cell line was selected as the best candidate for the ongoing experiments. Flow cytometry data confirmed that the immunophenotype of these cells is consistent with that of leukemic T-PLL cells. In addition, the comparable efficacy of BZ and CF demonstrated in the in vitro experiments with the SUP-T11 cell line and ex vivo T-PLL cells further supports the feasibility of using this model for future investigations.

## 4. Materials and Methods

### 4.1. Patient Recruitment and Screening at Diagnosis

This study comprised the enrolment of 17 patients diagnosed with T-PLL. For each patient, clinical data were collected at diagnosis and are summarized in Table 1. The availability of RNA-sequencing data from 10 T-PLL patients and 5 controls (CD4+ cells sorted from healthy donors; CTR) allowed their re-analysis to perform the drug screening test.

Peripheral blood mononuclear cells (PBMCs) of T-PLL patients were obtained after isolation with Ficoll-Hypaque (Sigma Aldrich, St. Louis, MO, USA) gradient centrifugation. The immunophenotype of the leukemic clone was determined by staining PBMCs with CD4, CD8, CD7, CD25, and CD26 antibodies and acquired with FACSCanto II (all Becton Dickinson, Franklin Lakes, NJ, USA). Since all cases were highly pure (>95% leukemic clone within the lymphocyte gate), no purification with immunomagnetic beads was required. To evaluate the presence of cell clonality, the T-Cell Receptor (TCR) Gamma Gene Rearrangement Assay (Invivoscribe, San Diego, CA, USA) was used. In addition, TCR-Vbeta expansion was evaluated with the IOTest Beta Mark Kit (Beckman Coulter, Brea, CA, USA). Sanger sequencing covering the hotspot regions of *STAT5B* (exons 14–16) and *JAK3* (exons 11–15), the most recurrent genetic lesions in T-PLL [9,13], was performed as previously reported [44] to investigate the mutational status of patients. This study and blood sample collection were approved by the Ethical Committee for Clinical Trials of Padova, while patients gave written informed consent according to the Helsinki Declaration prior to inclusion.

### 4.2. Drug Repurposing Screening

The drug screening was based on the Library of Integrated Cellular Signatures (LINCS) [45] program, which is accessible from the Broad Institute Connectivity Maps querying tools (https://clue.io accessed on 23 February 2023). LINCS includes transcriptomic signatures linked to genetic (CRISPR knock-out, shRNA knock-down, and ORF overexpression) and small-molecule (drugs and tool compounds) perturbations in fifty cell types of varied lineages. Individual LINCS members are associated with groups of compounds that share the same mechanism-of-action (MoA) classes.

The drug repurposing analysis was performed using QUADrATic (http://go.qub.ac.uk/QUADrATiC accessed on 23 February 2023) [46], focusing on the 300 genes (150 upregulated and 150 downregulated) most aberrantly expressed in CD4+ T-PLL patients (*n* = 10, Table 1) compared to normal counterparts (*n* = 5, CTR; CD4+ T-cells from healthy donors). This analysis was performed starting with RNA-sequencing data available in our laboratory for another ongoing project (manuscript in preparation). We retrieved the LINCS members most connected with the T-PLL gene expression signature and deemed the connections associated with an adjusted *p*-value of *q* < 0.05 (−10Log *q* threshold at 13) as significant, focusing particularly on negative connections.

### 4.3. In Vitro and Ex Vivo Cultures with Proteasome Inhibitors

Fresh or cryopreserved primary T-PLL cells were cultured at 2 × 10^6^ cells/mL in complete RPMI-1640 medium (EuroClone, Pero, Italy) supplemented with 10% fetal calf serum (Sigma-Aldrich), 2 mM glutamine, 100 U/mL penicillin, and 100 μg/mL streptomycin (EuroClone) and grown in 5% CO_2_ at 37 °C for 48 h. Cells were cultured with increasing doses of Bortezomib (BZ, Selleckchem), a reversible inhibitor of the 26S proteasome, and Carfilzomib (CF, Selleckchem), an irreversible PI, to study the effect of the PIs in inducing the apoptosis of leukemic cells. The concentration of BZ ranged between 2.6 nM and 7.8 nM, whereas for CF, the concentration ranged between 5 nM and 25 nM. Accordingly, cells without treatment were used as controls to determine the apoptotic basal level, and they were treated with the same concentration of DMSO that was used to resuspend both drugs to avoid bias in the analysis. The same experiments were conducted on the SUP-T11 cell line (Leibniz Institute DSMZ), which is considered the best in vitro model for studying T-PLL due to its molecular background, including overexpression of the *TCL1A* gene and distinctive surface markers. This is particularly relevant since a proper T-PLL cell line has not yet been established.

### 4.4. Evaluation of Apoptosis

Apoptosis was evaluated by staining 0.2 × 10^6^ primary T-PLL cells with anti-human CD7 (FITC) and PE-conjugated AnnexinV antibodies (BD) following the manufacturers’ protocol. The apoptosis was defined as the ratio of AnnexinV+ cells over the entire CD7+ T-lymphocyte population. For SUP-T11 cell line, the staining was performed only with PE-conjugated AnnexinV antibodies. All cytometric evaluations were performed with the BD FACS Canto II, and data were processed with Diva Software (BD, version 8.0.2).

In addition, apoptosis was confirmed by Western blot analysis. Cells were lysed in Sample Buffer (40 mM tris (hydroxymethyl) aminomethane, HCl pH 6.8, 7.5% glycerol, 1% sodium dodecyl sulfate). Total cell lysates were resolved in SDS-PAGE and transferred to a PVDF membrane. Blots were incubated with specific antibodies to evaluate the level of Poly (ADP-ribose) polymerase (PARP) and Caspase 9 (CASP9) protein cleavage (Aurogene) and detected using the ImageQuant LAS 500 (GE Healthcare, Milano, Italy). Protein cleavages were analyzed by calculating the ratio between the cleaved and uncleaved fractions for both proteins. In addition, GAPDH was used as the internal loading control. Densitometric analyses were performed using the ImageQuant TL software (version 8.1). All statistical analyses were performed with GraphPad software (version 8.0.2), and an ANOVA test was used to compare differences between conditions. *p* < 0.05 was considered to indicate a statistically significant difference.

## 5. Conclusions

While a more precise understanding of the specific mechanism of action of PIs in this context requires further investigation, our data strongly suggest that both Bortezomib and Carfilzomib significantly induce apoptosis of T-PLL cells. These preliminary results provide a solid rationale for incorporating PIs into the clinical management of T-PLL patients, thereby expanding treatment options and paving the way for more effective and potentially personalized treatment strategies for this aggressive leukemia with a poor prognosis. Finally, this study could serve as a starting point for evaluating other classes of compounds identified as being significant in the drug repurposing analysis, such as protein kinase C activators, tyrosine kinase inhibitors, and protease inhibitors. The ultimate goal is to increase the number of therapeutic options available for T-PLL patients.

## Figures and Tables

**Table 1:** Clinical data of the T-PLL cohort at diagnosis. Immunophenotype analysis was performed through flow cytometry (the positivity and negativity of the markers were determined based on the co-expression with CD4). Vβ expansion was considered absent (NE) when all the Vβ receptors recognized by the kit (about 70%) were expressed <5% in the lymphocyte gate. Sanger sequencing was performed covering the hotspot regions of each gene. Patients were subdivided according to the type of analysis performed (RNAseq and/or drug screening). F: female; M: male; WBCs: white blood cells (normal range: 3.5–11 × 10^9^/L); Lys: lymphocytes (%); Vβ: V beta expansion; NE: not expressed; NA: not available; wt: wild type.

TPLL	Sex	Age	WBCs	Lys	Immunophenotype	Vβ	STAT5B	JAK3	Analysis
1	M	86	NA	NA	CD4+ CD8− CD7+ CD25+ CD26−	NE	N642H	wt	RNAseq
2	F	57	50.3	90%	CD4+ CD8+ CD7+ CD25+ CD26+	NE	N642H	wt	RNAseq
3	F	65	229.2	80%	CD4+ CD8− CD7+ CD25+ CD26+	NE	N642H	wt	RNAseq
4	M	76	253.9	75%	CD4+ CD8− CD7+ CD25+ CD26+	13.1	Y665F	wt	RNAseq + drug screening
5	M	66	224.4	75%	CD4+ CD8− CD7+ CD25+ CD26+	7.1	N642H	wt	RNAseq + drug screening
6	F	80	290	88%	CD4+ CD8− CD7+ CD25+ CD26+	1	wt	wt	RNAseq
7	F	69	84.1	84%	CD4+ CD8− CD7+	NE	N642H	wt	RNAseq + drug screening
8	F	46	44.3	81%	CD4+ CD8− CD7+ CD25+ CD26+	5.3	wt	wt	RNAseq + drug screening
9	M	70	135.8	89%	CD4+ CD8− CD7+ CD25+ CD26+	7.1	wt	wt	RNAseq
10	M	70	305.7	96%	CD4+ CD8− CD7+ CD25+ CD26+	NE	wt	wt	RNAseq + drug screening
11	F	76	82.7	86%	CD4+ CD8− CD7+ CD25− CD26+	13.6	wt	wt	Drug screening
12	M	84	179.4	71%	CD4+ CD8− CD7+ CD25− CD26+	13.2	wt	wt	Drug screening
13	M	73	14.7	67%	CD4+ CD8− CD7+ CD25− CD26−	NE	wt	wt	Drug screening
14	M	88	10.4	72%	CD4+ CD8− CD7+ CD25+ CD26−	NE	wt	wt	Drug screening
15	M	69	16.2	54%	CD4+ CD8− CD7+ CD25− CD26−	7.2	wt	M511I	Drug screening
16	F	78	9.9	57%	CD4+ CD8− CD7+ CD25− CD26−	20	wt	M511I	Drug screening
17	F	76	74.1	NA	CD4+ CD8− CD7+ CD25− CD26−	NE	wt	wt	Drug screening

## Data Availability

Data contained within the article.
